# 

**Published:** 1902-01

**Authors:** 


### International Dental Journal

JAMES TRUMAN, D.D.S., Editor,
Philadelphia.

COLLABORATORS:

CHARLES P. BRIGGS, A.B., M.D., D.M.D., Boston.
JOHN A. McCLAIN, D.D.S., Philadelphia.
WILLIAM H. POTTER, A.B., D.M.D., Boston.
WM. LEON ELLERBECK, D.D.S., Philadelphia.

VOL. XXIII. JANUARY, 1902. No. 1.

THE INTERNATIONAL DENTAL PUBLICATION COMPANY,

227-231 SOUTH SIXTH ST., PHILADELPHIA.

EDITORIAL OFFICE, 4505 CHESTER AVENUE, PHILADELPHIA, PA.

$2.50 a Year, in Advance. Single Copies, 25 Cents.

PRINTED BY J. B. LIPPINCOTT COMPANY, PHILADELPHIA.

Entered at the Post-Office at Philadelphia, and admitted for transmission through the mails at second-class rates.

